# Identifying Components of a *Halobacterium salinarum* N-Glycosylation Pathway

**DOI:** 10.3389/fmicb.2021.779599

**Published:** 2021-12-02

**Authors:** Zlata Vershinin, Marianna Zaretsky, Ziqiang Guan, Jerry Eichler

**Affiliations:** ^1^Department of Life Sciences, Ben-Gurion University of the Negev, Be’er Sheva, Israel; ^2^Department of Biochemistry, Duke University Medical Center, Durham, NC, United States

**Keywords:** archaea, archaellin, dolichol phosphate, *Halobacterium salinarum*, N-glycosylation, S-layer glycoprotein

## Abstract

Whereas N-glycosylation is a seemingly universal process in Archaea, pathways of N-glycosylation have only been experimentally verified in a mere handful of species. Toward expanding the number of delineated archaeal N-glycosylation pathways, the involvement of the putative *Halobacterium salinarum* glycosyltransferases VNG1067G, VNG1066C, and VNG1062G in the assembly of an N-linked tetrasaccharide decorating glycoproteins in this species was addressed. Following deletion of each encoding gene, the impact on N-glycosylation of the S-layer glycoprotein and archaellins, major glycoproteins in this organism, was assessed by mass spectrometry. Likewise, the pool of dolichol phosphate, the lipid upon which this glycan is assembled, was also considered in each deletion strain. Finally, the impacts of such deletions were characterized in a series of biochemical, structural and physiological assays. The results revealed that VNG1067G, VNG1066C, and VNG1062G, renamed Agl25, Agl26, and Agl27 according to the nomenclature used for archaeal N-glycosylation pathway components, are responsible for adding the second, third and fourth sugars of the N-linked tetrasaccharide decorating *Hbt. salinarum* glycoproteins. Moreover, this study demonstrated how compromised N-glycosylation affects various facets of *Hbt. salinarum* cell behavior, including the transcription of archaellin-encoding genes.

## Introduction

N-glycosylation, a post-translational modification in which glycans are assembled on lipid carriers and then transferred to selected Asn residues in target proteins, is performed in all three domains of life (Aebi, 2013; Nothaft and Szymanski, 2013; Jarrell et al., 2014; Eichler, 2019). While the overall process shares similarities across evolution, various aspects of N-glycosylation are unique to Eukarya, Bacteria and Archaea. In the case of archaeal N-glycosylation, one finds degrees of diversity in terms of the lipid carrier upon which the N-linked glycan is assembled, the steps involved in N-linked glycan assembly and glycan composition and architecture not seen in the other two domains (Schwarz and Aebi, 2011; Eichler, 2013, 2020; Eichler and Guan, 2017). Such diversity is exemplified by N-glycosylation in *Halobacterium salinarum*, a halophilic archaeon that grow in NaCl concentrations near or at saturation (Grant et al., 2001). Although initially isolated from salted fish almost a century ago (Harrison and Kennedy, 1922), *Hbt. salinarum* first drew general attention with the 1971 discovery of bacteriorhodopsin, the light-driven proton pump isolated from purple membranes of this organism (Oesterhelt and Stoeckenius, 1971). *Hbt. salinarum* later provided the first example of protein glycoprotein outside Eukarya, with the surface (S)-layer glycoprotein that forms the S-layer surrounding the cell being shown to be N-glycosylated (Mescher and Strominger, 1976). Soon after, archaellins, the building blocks of the archaellar swimming device, the archaellum (Jarrell and Albers, 2012), were also shown to be N-glycosylated (Wieland et al., 1985).

Although limited by the tools available at the time, biochemical studies aimed at describing the pathways of *Hbt. salinarum* N-glycosylation conducted in 1980s were nonetheless able to provide a general outline of the N-glycosylation process (for review, see Lechner and Wieland, 1989). Such efforts identified aspects of the process not seen elsewhere at the time. For example, it was reported that both the S-layer glycoprotein and archaellins are modified by a sulfated tetrasaccharide initially assembled on a dolichol phosphate (DolP) lipid carrier, rather than the dolichol pyrophosphate (DolPP) used in eukaryotic N-glycosylation (Lechner et al., 1985a). Moreover, the DolP-linked glycan in this haloarchaeon was apparently modified by a peripherally attached 3-O-methylglucose not detected in the protein-linked glycan, suggestive of a unique mode of lipid-linked glycan translocation across the membrane; there, the 3-O-methylglucose is removed and the glycan is transferred to target Asn residues (Lechner et al., 1985b). Moreover, the S-layer glycoprotein was reported to be modified not only by that glycan derived from a DolP carrier but also by a second N-linked glycan derived from a DolPP carrier (Wieland, 1988). This second glycan, corresponding to 10–15 repeats of a sulfated pentasaccharide, was shown to be linked via N-acetylgalactosamine, rather than the N-acetylglucosamine employed in eukaryotic N-glycosylation (Paul et al., 1986). Still, none of the enzymes involved in N-linked glycan biosynthesis were identified at that time.

After a major gap of some 40 years, research into *Hbt. salinarum* N-glycosylation has resumed, this time incorporating bioinformatics and genetic tools, as well as other more modern approaches. Very recently, mass spectrometry was used to revisit the composition of the N-linked tetrasaccharide decorating the *Hbt. salinarum* S-layer glycoprotein and archaellins, showing it to comprise a hexose as the linking sugar, a sulfated hexuronic acid at position two, a hexuronic acid at position three and a second sulfated hexuronic acid at position four (Vershinin et al., 2021). In the same study, methylation of the fourth sugar of the tetrasaccharide at the DolP-linked stage was confirmed. Progress in deciphering the pathway used to assemble this N-linked tetrasaccharide has also been realized of late. Based on the ability of selected *Hbt. salinarum* genes to replace their counterparts known to participate in the assembly and attachment of an N-linked glycan in *Haloferax volcanii*, where N-glycosylation has been studied in detail (Eichler et al., 2013a; Jarrell et al., 2014), a putative *Hbt. salinarum* pathway used for biogenesis of the N-linked tetrasaccharide decorating the S-layer glycoprotein and archaellins was proposed (Kandiba and Eichler, 2015). After confirming the transcription of the genes encoding the components of this pathway, the same study also revealed VNG1048G and VNG1055G to act as a UDP-glucose dehydrogenase and a glucose-1-phosphate thymidyltransferase, respectively. In *Hfx. volcanii*, the homologs of these enzymes, i.e., AglM and AglF, cooperate to convert glucose-1-phosphate into UDP-glucuronic acid, the activated form of one of the sugars reportedly comprising the N-linked pentasaccharide decorating glycoproteins in this species (Yurist-Doutsch et al., 2010). More recently, the first direct support for the involvement of one of proteins assigned to this putative pathway in *Hbt. salinarum* N-glycosylation came with the demonstration of VNG1068G serving as the oligosaccharyltransferase AglB following deletion of the encoding gene and subsequent loss of N-glycosylation (Zaretsky et al., 2019).

In the present study, the involvement of other proteins comprising the putative *Hbt. salinarum* pathway responsible for the N-linked tetrasaccharide decorating glycoproteins in this haloarchaeon, specifically the putative glycosyltransferases VNG1062G, VNG1066C, and VNG1067G, was addressed.

## Materials and Methods

### Cell Growth

*Halobacterium salinarum* NRC-1 (ATCC strain 700922) parent strain and mutant strain cells were grown in medium containing 250 g NaCl, 20 g MgSO_4_⋅7H_2_O, 3 g sodium citrate, 2 g KCl, 10 g peptone per l, supplemented with 50 μg/ml uracil at 42^°^C (Darnell et al., 2017).

### Gene Deletions

*Halobacterium salinarum* Δ*ura3* cells deleted of *VNG1062G*, *VNG1066C*, and *VNG1067G* were generated as previously described, using the standard double-crossover counter-selection method (Peck et al., 2000; Zaretsky et al., 2019). Briefly, approximately 500 bp of flanking regions upstream and downstream of the target gene were PCR amplified (primers used are listed in Table 1) and inserted into the *Hin*dIII and *Nco*I restriction site of plasmid pNBK07 (Wilbanks et al., 2012) by isothermal assembly (Gibson et al., 2009) to create plasmid pNBK07ko. Following Sanger sequencing, plasmid pNBK07ko was introduced into the Δ*ura3* strain and selected on solid medium (20 g/l agar plates) containing mevinolin (10 μg/ml). The resulting strains were then counter-selected on plates containing 5-fluoroorotic acid (300 μg/ml) and uracil to remove the integrated plasmid, yielding the deletion strains. All incubation steps during transformation and counter-selection were conducted at 42°C. Deletions were confirmed by PCR and qRT-PCR (Supplementary Figure 1). PCR amplification of genomic DNA was performed using primers directed against sequences up- and down stream of the deleted gene. The resulting products were validated by PCR. qRT-PCR was performed as previously described (Zaretsky et al., 2019), with *vngRS02595* (formerly *vng0657G*) serving as the reference gene (Darnell et al., 2020). Primers used for PCR and qRT-PCR are listed in Table 1.

**TABLE 1:** Primers used in this study.

Primer name	Sequence
**Deletion primers**	
VNG1062-up-F	AGAAGCGAGGAAGTCCCAGGTAG
VNG1062-up-R	CCCAACCTCTGACATCAGGTCGAGGCATTC GTCACGCCACGAGC
VNG1062-down-F	GCTCGTGGCGTGACGAATGCCTCGACCTGATG TCAGAGGTTGGG
VNG1062-down-R	CTGGGATAGCGAACTCCGTGTC
VNG1062-Hind-Gibs-F	GAGCAGACGCATCTGGATCCACGAAGCTT CAGAAGCGAGGAAGT CCCAGGTAG
VNG1062-Nco-Gibs-R	AGGTATCTAGAACCGGTGACGTCACCATGGCT GGGATAGCGAACT CCGTGTC
VNG1066-up-F	TCG GAT GAC GTG TTA TGG GAT ATC
VNG1066-up-R	GCCGAACCTCGTGGCTAACTGGGTCATCT ACTGTAGCAAGTACGTG
VNG1066-down-F	CACGTACTTGCTACAGTAGATGACCCAGTTAG CCACGAGGTTCGGC
VNG1066-down-R	GACTTCTTCCCCGCCGAGTTC
VNG1066-Hind-Gibs-F	TCGAGCAGACGCATCTGGATCCACGAAGCTT CTCGGATGACGTGTT ATGGGATATC
VNG1066-Nco-Gibs-R	GGTATCTAGAACCGGTGACGTCACCATGGCGA CTTCTTCCCCGCCG AGTTC
VNG1067-up-F	CCAGAATGACCGCTCCAAAGAGG
VNG1067-up-R	GACATCCCCGCGCCTACGAGGGGCATCCGGT CCGACC
VNG1067-down-F	GGTCGGACCGGATGCCCCTCGTAGGCGCGGG GATGTC
VNG1067-down-R	TCGACTACCTCGGCTGCAAC
VNG1067-Hind-Gibs-F	CGAGCAGACGCATCTGGATCCACGAAGCTTCC CAGAATGACCGCT CCAAAGAGG
VNG1067-Nco-Gibs-R	AGGTATCTAGAACCGGTGACGTCACCATGGC GTCGACTA CCTCGGCTGCAAC
fr-pNKB07-seq	TGTCACAGACGACGCTCCCGCA
rev-pNBK07-seq	GTTGGGTAACGCCAGGGTTTTC
**qPCR primers**	
VNG1062G FW	GGATGAGACGATGCAAGTGATA
VNG1062G Rev	AGCTCTGAATCTCGGTCTCT
VNG1066C FW	AACTAACGTGCCCTGAAGAC
VNG1066C Rev	GAACACCTTACGACGGACAA
VNG1067G FW	AACAGCTCCTCGGTGTCTA
VNG1067G Rev VNGRS02595 FW VNGRS02595 Rev	GGGAAGAAGTCGGGTTTCTG CGGATTCGGTCGAGTTTCAT CACATCGTGGTGATCCAGTT
FlaA1 FW	CAAGACCGCTAGTGGGACC
FlaA1 Rev	GCGTCGGCAGTGCTACC
FlaA2 FW	ACCCTAACGCACGCCAAC
FlaA2 Rev	CGTTGTCGTTGTTCCCCTTG
FlaB1 FW	CGAATCCATCAAGGGCAGC
FlaB1 Rev	GCTGCACCTCGTCACCAG
FlaB2 FW	GAATTCGATTAAGGGCGACAAC
FlaB2 Rev	CAGTCCATTGGTGGTGATCT
FlaB3 FW	CTCACGAAATCCACGATCCA
FlaB3 Rev	TGATGGATTCGGTGGTGAAG

### Enrichment of Archaellins

The five *Hbt. salinarum* archaellins (FlaA1, FlaA2, FlaB1, FlaB2, and FlaB3) were enriched from spent growth medium as previously described (Zaretsky et al., 2019). Briefly, cultures were grown to logarithmic (OD_600_ ∼ 0.8) phase and held at room temperature without shaking for 24 h. The cultures were centrifuged for 30 min (6,000 × *g*, 15°C). The supernatant (post-spin 1 supernatant) was collected and centrifuged again for 15 min (16,000 × *g*, 15°C). The supernatant (post-spin 2 supernatant) was centrifuged for 2 h (40,000 × *g*, 4°C). The pelleted material (post-spin 3) was resuspended by shaking in 1 ml of 4 M basal salt solution (250 g NaCl, 20 g MgSO_4_⋅7H_2_O, 3 g sodium citrate, 2 g KCl per l) and heated for 10 min at 90°C. The heated suspension was centrifuged for 15 min (16,000 × *g*, 15°C). The resulting supernatant (post-spin 4 supernatant) was maintained at 4°C for 24 h and centrifuged for 2 h (40,000 × *g*, 4°C). After removal of the supernatant (post-spin 5 supernatant), the pellet (post-spin 5 pellet) was resuspended in sample buffer and separated by 12% SDS-PAGE and stained with Coomassie InstantBlue (Expedeon).

### Preparation of Total *Halobacterium salinarum* Lipid Extracts

The total lipid contents of *Hbt. salinarum* parent and deletion stain cells were extracted using the protocol described by Kelleher et al. (2001), with minor modifications. *Hbt. salinarum* cells were harvested (8,000 × g, 30 min, 4^°^C), the pellet was resuspended in 2 M NaCl, 50 mM Tris–HCl, pH 7.2, centrifuged again (8,000 × *g*, 90 min, 4^°^C) and the pellet was frozen at −20^°^C until extraction was performed. At that point, the pelleted cells (∼15 g) were thawed, resuspended in 20 ml double-distilled water (DDW) and DNase (1.7 μg/ml; Sigma, St. Louis, MO, United States) and stirred overnight at room temperature, followed by sonication on ice at room temperature (2 s on, 5 s off, for a total of 1 min; Vibracell VCX750 ultrasonic cell disrupter, Sonics, Newtown, CT, United States). After sonication, the cell suspension was centrifuged for 30 min at 11,000 rpm in a SW-40 rotor (Beckman Coulter) at 4°C to clear non-broken cells and other debris. The supernatant was transferred into fresh tubes and centrifuged for an additional 45 min at 36,000 rpm in a SW-40 rotor at 4°C. The resulting supernatant was removed and the pellet was re-suspended in 35 ml homogenization buffer (150 mM NaCl, 50 mM Tris–HCl, pH 8) containing 50 ml CHCl_3_:CH_3_OH (3:2) at 4°C and homogenized using a Pyrex Potter-Elvehjem tissue grinder (Thomas Scientific). After homogenization, 65 ml cold (4°C) CHCl_3_:CH_3_OH (3:2) were added and the homogenate was mixed by vigorous shaking before centrifugation (3,400 × *g*, 15 min, 4°C). The resulting clear upper aqueous and lowest organic phases were removed and the middle (solid) phase was resuspended in 75 ml of CHCl_3_:CH_3_OH (3:2) containing 1 mM MgCl_2_ at room temperature. After vigorous re-homogenization, the suspension was adjusted to a total volume of 150 ml and centrifuged for 15 min (3,400 × *g*, 4°C). The supernatant was removed and the pellet was suspended in 150 ml CH_3_OH containing 4 mM MgCl_2_ before centrifugation (3,400 × *g*, 15 min, 4°C). These steps were repeated and the resulting pellet was suspended in 150 ml CHCl_3_:CH_3_OH:DDW (10:10:3) and centrifuged in a swing-out rotor (1,000 rpm) for 15 min at 22°C. The supernatant was removed and stored while the pellet were re-extracted with 100 ml CHCl_3_:CH_3_OH:DDW (10:10:3) at 37°C and centrifuged as above. The supernatants obtained from the first and second extractions were combined, filtered through glass wool and the ensuing solution was evaporated at 30°C. Thereafter, any remaining solvents were removed using a stream of nitrogen.

### Liquid Chromatography-Electrospray Ionization Mass Spectrometry (LC-ESI MS)

Normal phase LC-ESI/MS of *Hbt. salinarum* lipids was performed using an Agilent 1200 Quaternary LC system coupled a high-resolution TripleTOF5600 mass spectrometer (Sciex, Framingham, MA, United States). An Ascentis Si HPLC column (5 μm, 25 cm × 2.1 mm) was used. Mobile phase A consisted of chloroform/methanol/aqueous ammonium hydroxide (800:195:5, v/v/v). Mobile phase B consisted of chloroform/methanol/water/aqueous ammonium hydroxide (600:340:50:5, v/v/v/v). Mobile phase C consisted of chloroform/methanol/water/aqueous ammonium hydroxide (450:450:95:5, v/v/v/v). The elution program consisted of the following: 100% mobile phase A was held isocratically for 2 min and then linearly increased to 100% mobile phase B over 14 min and held at 100% B for 11 min. The LC gradient was then changed to 100% mobile phase C over 3 min and held at 100% C for 3 min, and finally returned to 100% A over 0.5 min and held at 100% A for 5 min. The total LC flow rate was 300 μl/min. The post-column splitter diverted ∼10% of the LC flow to the ESI source of the TripleTOF5600 XL mass spectrometer, with MS settings as follows: IS = −4500 V, CUR = 20 psi, GS1 = 20 psi, DP = −55 V, and FP = −150 V. For MS/MS, collision-induced dissociation (CID) was performed with collision energy ranging from 40 to 70 V (laboratory frame of energy) and with nitrogen as the collision gas. Data acquisition and analysis were performed using the instrument’s Analyst QS software.

For LC-ESI MS analysis, the *Hbt. salinarum* S-layer glycoprotein, recognized via its unique SDS-PAGE migration pattern, was subjected to in-gel digestion, as were the isolated archaellins. S-layer glycoprotein- and archaellin-containing bands were excised from SDS-PAGE gels using a clean scalpel, destained in 400 μl of 50% (vol/vol) acetonitrile (Sigma) in 40 mM NH_4_HCO_3_, pH 8.4, dehydrated with 100% acetonitrile, and dried using a SpeedVac drying apparatus. The proteins in the gel slices were reduced with 10 mM dithiothreitol (Sigma) in 40 mM NH_4_HCO_3_ at 56°C for 60 min and then alkylated for 45 min at room temperature with 55 mM iodoacetamide in 40 mM NH_4_HCO_3_. The gel pieces were washed with 40 mM NH_4_HCO_3_ for 15 min, dehydrated with 100% acetonitrile, and SpeedVac dried. The gel slices were rehydrated with 12.5 ng/μl of mass spectrometry (MS)-grade Trypsin (Thermo Scientific) in 40 mM NH_4_HCO_3_ and incubated overnight at 37°C. The protease-generated peptides were extracted with 0.1% (v/v) formic acid in 20 mM NH_4_HCO_3_, followed by sonication for 20 min at room temperature, dehydration with 50% (v/v) acetonitrile, and additional sonication. After three rounds of extraction, the gel pieces were dehydrated with 100% acetonitrile and dried completely with a SpeedVac. In some cases, 12.5 ng/μl Glu-C (V8) protease (Promega, sequencing-grade) in 40 mM NH_4_HCO_3_ were added and the samples were incubated overnight at 37°C. After three rounds of extraction, the gel pieces were dehydrated with 100% acetonitrile and dried completely with a SpeedVac. Both trypsin- and trypsin and Glu-C-treated samples were resuspended in 5% (v/v) acetonitrile containing 1% formic acid (v/v) and infused into the mass spectrometer using static nanospray Econotips (New Objective, Woburn, MA, United States). The protein digests were separated on-line by nano-flow reverse-phase liquid chromatography (LC) by loading onto a 150-mm by 75-μm (internal diameter) by 365-μm (external diameter) Jupifer pre-packed fused silica 5-μm C_18_ 300Å reverse-phase column (Thermo Fisher Scientific, Bremen, Germany). The sample was eluted into the LTQ Orbitrap XL mass spectrometer (Thermo Fisher Scientific) using a 60-min linear gradient of 0.1% formic acid (v/v) in acetonitrile/0.1% formic acid (1:19, v/v) to 0.1% formic acid in acetonitrile/0.1% formic acid (4:1, v/v) at a flow rate of 300 nl/min. A full scan, acquired at 60,000 resolution, was followed by CID MS/MS analysis performed for the five most abundant peaks, in a data–dependent mode. Fragmentation (with minimum signal trigger threshold set at 500) and detection of fragments were carried out in a linear ion trap. Maximum ion fill time settings were 500 ms for a high–resolution full scan in an Orbitrap analyzer and 200 ms for MS/MS analysis in the ion trap. The AGC settings were 5 × 10^5^ and 1 × 10^4^ (MS/MS) for Orbitrap and linear ion trap analyzers, respectively. Mass tolerance for precursors and fragmentations was set to 10 ppm and 0.8 Da, respectively. Glycopeptides were manually identified using the instrument’s Analyst QS software and based on the information of high-resolution accurate mass measurement and MS/MS fragmentation.

### Proteolytic Digestion of the S-Layer

To assess how the deletion of genes encoding components of a putative N-glycosylation pathway affected resistance of the S-layer to proteolytic attack, *Hbt. salinarum* cells (1 ml) of the parent and deletion strains were grown to OD_600_ = 1.0 and challenged with proteinase K (1 mg/ml, final concentration; Sigma) at 37^°^C. Aliquots (100 μl) were removed immediately prior to incubation with proteinase K (considered as the *t* = 0 point) and at 30 min intervals following addition of the protease for up to 150 min. Following separation by 8% SDS-PAGE, the proteins were Coomassie-stained. The levels of S-layer glycoprotein were then considered.

### Plate Motility Assay

To assay motility, parent and deletion stain cells were grown on semi-solid medium containing 0.3% agar (w/v). Aliquots (10 μl) of liquid cultures of the parent or mutant strains grown to logarithmic phase (OD_600_ ∼ 0.8) were placed at the center of the agar surface. The plates were incubated for 3 days at 42°C (Patenge et al., 2001), after which time the diameter of the motility halo was measured. Where the halos were not perfectly circular, the diameter was considered as the average of the longest and shortest linear spans of the halo area. Three plates each were assessed per strain type. To confirm the viability of each strain after the 3 day-long period of incubation, cells from each plate were picked and grown for 4 days at 42°C in 10 ml of growth medium.

## Results

### Reduced *Halobacterium salinarum* Growth, S-Layer Glycoprotein Protease Resistance, Motility and Archaellin Levels Are Seen in the Absence of VNG1062G, VNG1066C or VNG1067G

To date, the only protein shown to participate in *Hbt. salinarum* N-glycosylation is VNG1068G, which corresponds to the archaeal oligosaccharyltransferase AglB (Zaretsky et al., 2019). However, based on their abilities to replace components of the well-characterized *Hfx. volcanii* N-glycosylation pathway, putative N-glycosylation pathway functions have been assigned to other *Hbt. salinarum* proteins, including the predicted glycosyltransferases VNG1062G, VNG1066C, and VNG1067G (Kandiba and Eichler, 2015). As a first step in determining whether VNG1062G, VNG1066C or VNG1067G indeed contribute to *Hbt. salinarum* N-glycosylation, cells lacking the encoding genes were assessed in assays in which cells lacking AglB, and hence unable to N-glycosylate, behaved differently than did parent strain cells (Zaretsky et al., 2019).

**FIGURE 1:**
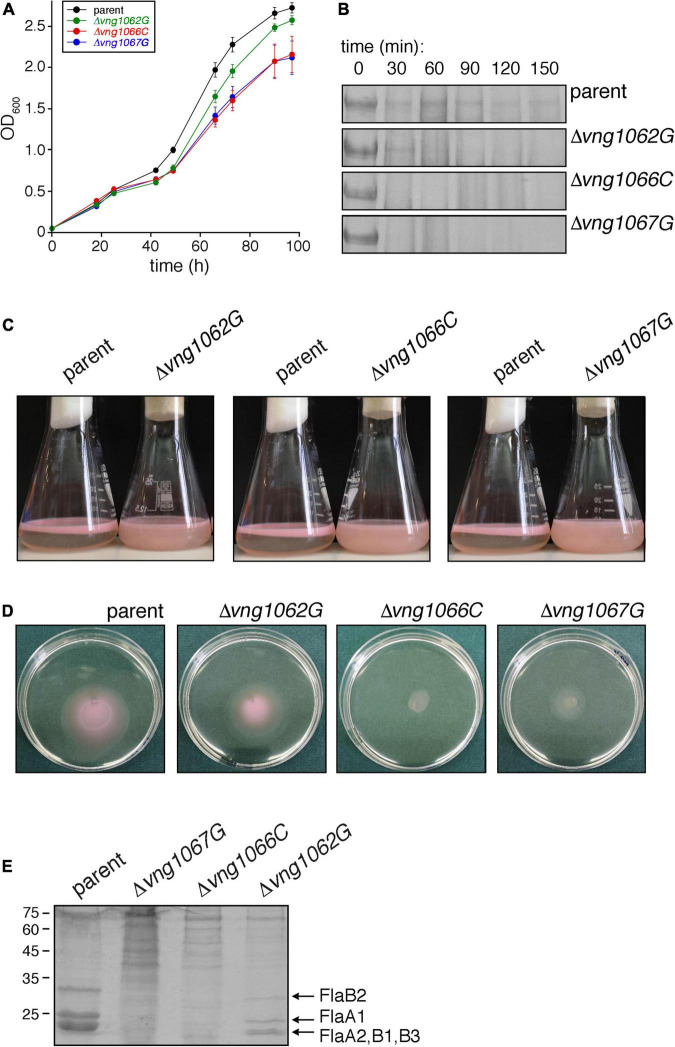
The absence of VNG1062G, VNG1066C or VNG1067G affects various aspects of *Hbt. salinarum* behavior. **(A)** Growth of Δ*vng1062G*, Δ*vng1066C*, and Δ*vng1067G* strains is compromised, relative to the parent strain. Each point represents the average of three biological repeats ± standard error of the mean. **(B)** Exposure of the S-layer of the Δ*vng1062G*, Δ*vng1066C*, and Δ*vng1067G* strains to added proteinase K leads to more rapid digestion of the S-layer glycoprotein, relative to what occurs when the parent strain is similarly treated. Shown are regions of 8% SDS-PAGE gels containing the Coomassie-stained S-layer glycoprotein found in the different cultures challenged with the protease for the indicated intervals. **(C)** Stationary phase cells of the parent, Δ*vng1062G*, Δ*vng1066C*, and Δ*vng1067G* strains were left unperturbed overnight. Cells of the mutant cultures remain dispersed in the medium, whereas cells of the parent strain collect at the medium-air interface. **(D)** Cell motility is compromised in the Δ*vng1062G*, Δ*vng1066C*, and Δ*vng1067G* strains. Aliquots (10 μl) of liquid cultures of the parent and mutant strains in logarithmic phase were applied to the center of Petri dishes containing solid medium that included 0.3% agar (w/v). After 3 days at 42°C, the diameter of the halo that appeared was measured. Where the halos were not perfectly circular, the diameter was considered as the average of the longest and shortest linear spans of the halo area. Three plates each were assessed per strain. A representative set of plates is presented. **(E)** Lower amounts of archaellins are found in the spent growth media of the Δ*vng1062G*, Δ*vng1066C*, and Δ*vng1067G* strains than of the parent stain. Archaellins were isolated as described in the “Materials and Methods,” separated by SDS-PAGE and Coomassie-stained. The positions of molecular weight markers are indicated on the left, while the different archaellins are indicated on the right.

Initially, the effects of *vng1062G, vng1066C* or *vng1067G* deletion on cell growth was considered. Such analysis revealed that the Δ*vng1066C* and Δ*vng1067G* strains not only grew slower than did the parent strain but that they also failed to reach the level of growth of the parent strain. At the same time, the Δ*vng1062G* strain grew slightly more slowly than the parent strain yet essentially reached the same level of growth (Figure 1A). Hence, as previously seen with Δ*aglB* cells (Zaretsky et al., 2019), the absence of VNG1062G, VNG1066C or VNG1067G comprise *Hbt. salinarum* cell growth, albeit to differing degrees.

A second assay addressed how the absence of VNG1062G, VNG1066C or VNG1067G affected susceptibility of the S-layer glycoprotein, the major glycoprotein in *Hbt. salinarum*, to added protease. Accordingly, cells of the parent and mutant strains were challenged with proteinase K over 2.5 h and the amount of remaining S-layer glycoprotein in aliquots removed every 30 min was revealed by SDS-PAGE and Coomassie staining (Figure 1B). Whereas a considerable amount of S-layer glycoprotein disappeared from parent strain cells after 30 min of proteolytic digestion, this level did not significantly change over the next 2 h of exposure to the protease. By contrast, the same protein in the Δ*vng1066C* and Δ*vng1067G* cells was almost fully digested within the first 30 min after addition of protease. In the case of Δ*vng1062G* cells, although considerable digestion of the S-layer glycoprotein occurred within the first 30 min of the protease treatment, the protein could still be clearly seen after 90 min of proteolysis. As such, it appears that the absence of VNG1062G, VNG1066C or VNG1067G renders the S-layer glycoprotein more susceptible to proteolytic attack (again, to different degrees), most likely by affecting architecture of the S-layer comprised of this glycoprotein.

Efforts next focused on the interplay between archaellin N-glycosylation and cell motility, given how previous studies demonstrated that cells lacking AglB were non-motile (Zaretsky et al., 2019). Accordingly, the ability of cells of the Δ*vng1062G*, Δ*vng1066C*, and Δ*vng1067G* strains to migrate to the surface of the growth medium in standing stationary-phase cultures was assessed. Unlike parent stain cells, which collect at the liquid-air interphase in the culture vessels, cells of the mutant strain cultures instead remained dispersed throughout the growth medium (Figure 1C), as previously seen with cells of the Δ*aglB* strain (Zaretsky et al., 2019). However, as closer examination of the standing cultures revealed slight differences in the colors of the three mutant strains, possibly reflecting some degree of deletion strain-specific cell motility, a second, more quantitative motility assay was performed. In this assay, the impacts of *vng1062G, vng1066C* or *vng1067G* deletion on motility were assessed by following cell migration on soft agar plates, as previously described (Zaretsky et al., 2019). In this assay, 10 μl aliquots of a parent strain culture or of the Δ*vng1062G*, Δ*vng1066C* or Δ*vng1067G* strain cultures were spotted onto the center of soft agar plates. After 3 days at 42°C, clear differences in the areas covered by the cells of each strain were apparent (Figure 1D). Specifically, cells of the parent strain had expanded to cover an area with a diameter of 4.23 ± 0.04 mm (average of three plates ± standard error of the mean). Under the same conditions, cells of the Δ*vng1062G*, Δ*vng1066C*, and Δ*vng1067G* strains yielded areas with diameters of 3.7 ± 0.25 mm, 1.3 ± 0.09 mm, and 2.35 ± 0.03 mm, respectively. The subsequent growth of cells from each strain following their transfer from the agar plates into liquid medium confirmed that the differential migration observed was not due to cell death (not shown). Thus, based on these two motility assays, it can be concluded that in the absence of VNG1062G, VNG1066C or VNG1067G, *Hbt. salinarum* cell motility is decreased, once again to different extents.

Finally, whereas considerable amounts of archaellins could be isolated from the spent growth medium of parent strain cells, far lower amounts of archaellins were enriched from the growth medium of deletion strain cells (Figure 1E). Specifically, only low levels of FlaA1, FlaA2, FlaB1, FlaB2, and FlaB3 were detected in the spent medium of Δ*vng1066C* and Δ*vng1067G* cells. While far more archaellins could be isolated from the spent medium of Δ*vng1062G* strain cells, these were well below what was seen in the medium of parent strain cells. Thus, the absence of VNG1062G, VNG1066C or VNG1067G greatly compromised the amount of archaellins found in the spent growth medium, relative to the parent strain, with the absence of VNG1062G having less of an effect than the absence of VNG1066C or VNG1067G.

### The Absence of VNG1062G, VNG1066C or VNG1067G Results in Compromised N-Glycosylation

The involvement of VNG1062G, VNG1066C or VNG1067G in *Hbt. salinarum* N-glycosylation was next directly tested by describing the effects of deleting each encoding gene on the N-linked tetrasaccharide that decorates glycoproteins in this haloarchaeon (Vershinin et al., 2021). For this, the composition of the N-linked tetrasaccharide attached to modified Asn residues was examined by LC-ESI MS following proteolytic digestion of *Hbt. salinarum* glycoproteins.

**FIGURE 2:**
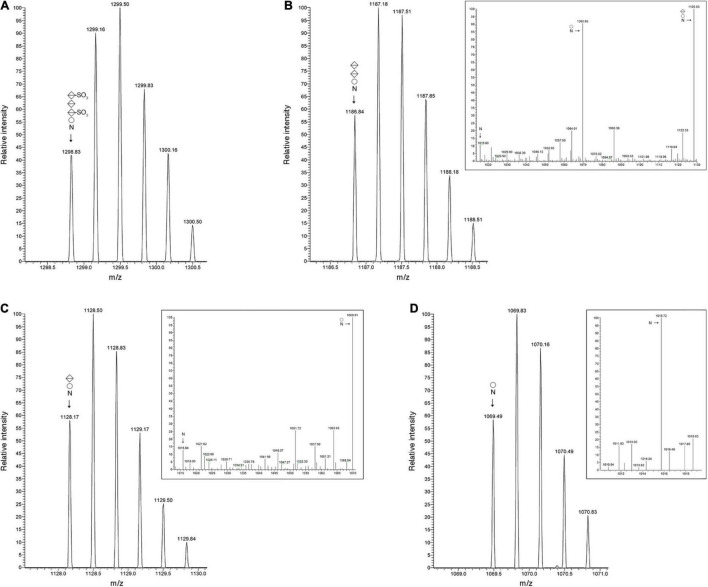
S-layer glycoprotein N-glycosylation is compromised in cells of the Δ*vng1062G*, Δ*vng1066C*, and Δ*vng1067G* strains. **(A)** LC-ESI MS profile of an S-layer glycoprotein Asn-479-containing peptide from parent strain cells modified by a tetrasaccharide comprising a hexose, a sulfated hexuronic acid, a hexuronic acid and a sulfated hexuronic acid. **(B)** In Δ*vng1062G* strain cells, the same fragment is only modified by the first three sugars of the tetrasaccharide, with no sulfation of the hexuronic acid at position two. The MS/MS profile of this peptide reveals breakdown products corresponding to the same peptide modified by the first two sugars and the first sugar of the tetrasaccharide and by no sugars (insert). **(C)** In Δ*vng1066C* strain cells, the same fragment is only modified by the first two sugars of the tetrasaccharide, with no sulfation of the hexuronic acid at position two. The MS/MS profile of this peptide reveals breakdown products corresponding to the same peptide modified by the first sugar and by no sugars (insert). **(D)** In Δ*vng1067G* strain cells, the same fragment is only modified by the first sugar of the tetrasaccharide. The MS/MS profile of this peptide reveals a breakdown product corresponding to the same peptide not modified by sugars (insert). In each panel, circles correspond to hexoses, split diamonds correspond to hexuronic acids and the N corresponds to the Asn-479-containing peptide.

The *Hbt. salinarum* S-layer glycoprotein is modified at ten of twelve potential N-glycosylation sites, including Asn-479, by a tetrasaccharide recently redefined as comprising a hexose as the linking sugar, a sulfated hexuronic acid at position two, a hexuronic acid at position three and a second sulfated hexuronic acid at position four (Lechner and Sumper, 1987; Vershinin et al., 2021). Accordingly, LC-ESI MS of the S-layer glycoprotein-derived ^475^SDAVNSSGGVKDNIDTSDFNQGVSSTSSIR^504^ peptide yielded an MS profile that included a peak of *m/z* 1298.83, corresponding to the [M + 3H]^3+^ ion of this Asn-479-containing peptide modified by a tetrasaccharide comprising a hexose, a hexuronic acid and two sulfated hexuronic acids ([M + 3H]^3+^ ion calculated *m/z* 1298.82) (Figure 2A). When the MS profile of the same peptide from Δ*vng1062G*, Δ*vng1066C* or Δ*vng1067G* cells was considered, no peaks corresponding to the tetrasaccharide-modified peptide were observed. Instead, the MS profiles from the mutant strains included peaks corresponding to the trypsin-generated Asn-479-containing peptide only modified by precursors of the N-linked tetrasaccharide. In the Δ*vng1062G* strain, this corresponded to a peak of *m/z* 1186.84, consistent with the peptide modified with a hexose and two hexuronic acids ([M + 3H]^3+^ ion calculated *m/z* 1186.81) (Figure 2B). The MS/MS profile of this peak revealed a breakdown pattern that included fragments of *m/z* 1128.53, 1069.85, and 1015.80 respectively consistent with the peptide modified with a hexose and a hexuronic acid ([M + 3H]^3+^ ion calculated *m/z* 1128.14), with a hexose ([M + 3H]^3+^ ion calculated *m/z* 1069.47), and not modified with any sugar ([M + 3H]^3+^ ion calculated *m/z* 1015.47) (Figure 2B, inset). The Δ*vng1066C* strain included a peak of *m/z* 1128.17, consistent with the peptide modified with a hexose and a hexuronic acid ([M + 3H]^3+^ ion calculated *m/z* 1128.14) (Figure 2C). The MS/MS profile of this peak revealed a breakdown pattern that included fragments of *m/z* 1069.91 and 1015.84, respectively consistent with the peptide modified with a hexose ([M + 3H]^3+^ ion calculated *m/z* 1069.47) and not modified with any sugar ([M + 3H]^3+^ ion calculated *m/z* 1015.47) (Figure 2C, inset). Finally, the Δ*vng1067G* strain presented a peak of *m/z* 1069.49, consistent with the peptide modified with a hexose ([M + 3H]^3+^ ion calculated *m/z* 1069.47) (Figure 2D). The MS/MS profile of this peak revealed a breakdown pattern that included a fragment of *m/z* 1015.72, consistent with the unmodified peptide ([M + 3H]^3+^ ion calculated *m/z* 1015.47) (Figure 2D, inset). An additional comparison of the MS profiles of the Asn-479-containing peptide from the parent and mutant strains is presented in Supplementary Figure 2A.

To examine whether *vng1062G*, *vng1066C* or *vng1067G* deletion also compromised archaellin N-glycosylation, LC-ESI MS was performed on archaellins isolated from the spent growth medium of parent strain cells and of each mutant strain. Much as seen with the N-glycosylation profile of the S-layer glycoprotein from the mutant strains, proteolytic digestion with trypsin and Glu-C of FlaA1, FlaA2, FlaB1, FlaB2, and FlaB3 yielded glycopeptides that included Asn residues modified by precursors of the N-linked tetrasaccharide decorating these residues in the same proteins isolated from parent strain cells. Specifically, the Δ*vng1062G* strain only presented glycopeptides modified with the precursor containing a hexose and two hexuronic acids, the Δ*vng1066C* strain only presented glycopeptides modified with the precursor containing a hexose and a hexuronic acid and the Δ*vng1067G* strain only presented glycopeptides modified with the precursor containing a hexose.

**FIGURE 3:**
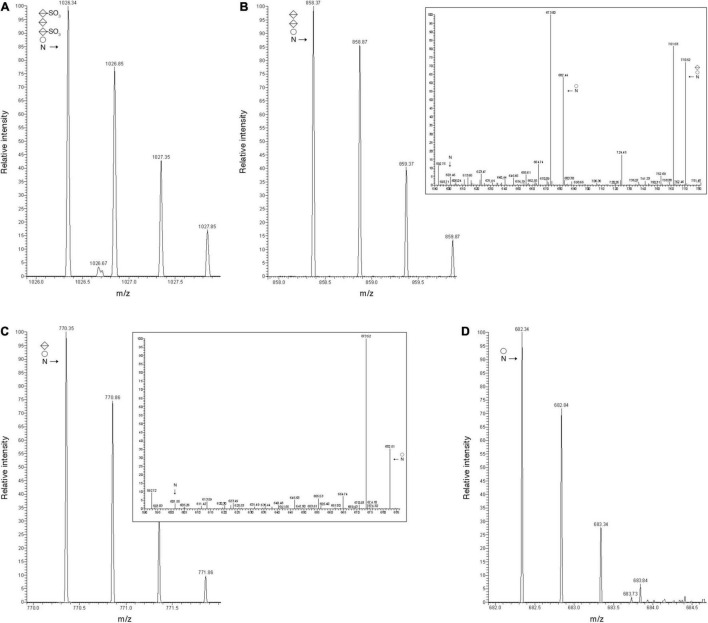
Archaellin N-glycosylation is compromised in cells of the Δ*vng1062G*, Δ*vng1066C*, and Δ*vng1067G* strains. **(A)** LC-ESI MS profile of the protease-generated peptide QAAGADNINLSK common to the archaellins FlaA1, FlaA2, and FlaB2 in parent strain cells is modified by a tetrasaccharide comprising a hexose, a sulfated hexuronic acid, a hexuronic acid and a sulfated hexuronic acid. **(B)** In Δ*vng1062G* strain cells, the same fragment is only modified by the first three sugars of the tetrasaccharide, with no sulfation of the hexuronic acid at position two. The MS/MS profile of this peptide reveals breakdown products corresponding to the same peptide modified by the first two sugars and the first sugar and by no sugars (insert). **(C)** In Δ*vng1066C* strain cells, the same fragment is only modified by the first two sugars of the tetrasaccharide, with no sulfation of the hexuronic acid at position two. The MS/MS profile of this peptide reveals breakdown products corresponding to the same peptide modified by the first sugar and by no sugars (insert). **(D)** In Δ*vng1067G* strain cells, the same fragment is only modified by the first sugar of the tetrasaccharide. In each panel, circles correspond to hexoses, split diamonds correspond to hexuronic acids and the N corresponds to the Asn-479-containing peptide.

The effects of these gene deletions are exemplified by the N-glycosylation profiles of the protease-generated peptide common to FlaA1, FlaA2, and FlaB2 (QAAGADNI**N**LSK, with the modified Asn residue in bold) from the parent and mutant strains. The MS profile of the parent strain-derived peptide included a peak of *m/z* 1026.34, corresponding to the [M + 2H]^2+^ ion of this peptide modified by a tetrasaccharide comprising a hexose, a hexuronic acid and two sulfated hexuronic acids ([M + 2H]^2+^ ion calculated *m/z* 1026.31) (Figure 3A). At the same time, the MS profiles from the mutant strains included peaks corresponding to the same peptide solely modified by precursors of this tetrasaccharide. This corresponded to a peak of *m/z* 858.37 in the Δ*vng1062G* strain MS profile, consistent with the peptide modified with a hexose and two hexuronic acids ([M + 2H]^2+^ ion calculated *m/z* 858.31) (Figure 3B). The MS/MS profile of this peak revealed a breakdown pattern that included fragments of *m/z* 770.52, 682.44, and 601.46, respectively consistent with the peptide modified with a hexose and a hexuronic acid ([M + 2H]^2+^ ion calculated *m/z* 770.31), with a hexose ([M + 2H]^2+^ ion calculated *m/z* 682.31), and not modified with any sugar ([M + 2H]^2+^ ion calculated *m/z* 601.31) (Figure 3B, inset). The Δ*vng1066C* strain included a peak of *m/z* 770.35, consistent with the peptide modified with a hexose and a hexuronic acid ([M + 2H]^2+^ ion calculated *m/z* 770.31) (Figure 3C). The MS/MS profile of this peak revealed a breakdown pattern that included fragments of *m/z* 682.51 and 601.55, respectively consistent with the peptide modified with a hexose ([M + 2H]^2+^ ion calculated *m/z* 682.31) and not modified with any sugar ([M + 2H]^2+^ ion calculated *m/z* 601.31) (Figure 3C, inset). Finally, the Δ*vng1067G* strain presented a peak of *m/z* 682.34, consistent with the peptide modified with a hexose ([M + 2H]^2+^ ion calculated *m/z* 682.31) (Figure 3D). An additional comparison of the MS profiles of this archaellin-derived peptide from the parent and mutant strains is presented in Supplementary Figure 2B.

The deletion of *vng1062G*, *vng1066C* or *vng1067G* had similar effects on the same N-tetrasaccharide also found on the FlaA1 glycopeptide TASGTDTVDYANLTVR, the FlaA2 glycopeptide FNTTSIK, the FlaB2 glycopeptide VVNYANLTVR and the FlaA2/B1/B3 glycopeptide VDYVNLTVR (not shown).

### VNG1062G, VNG1066C, and VNG1067G Contribute to Tetrasaccharide Assembly on a Dolichol Phosphate Carrier

**FIGURE 4:**
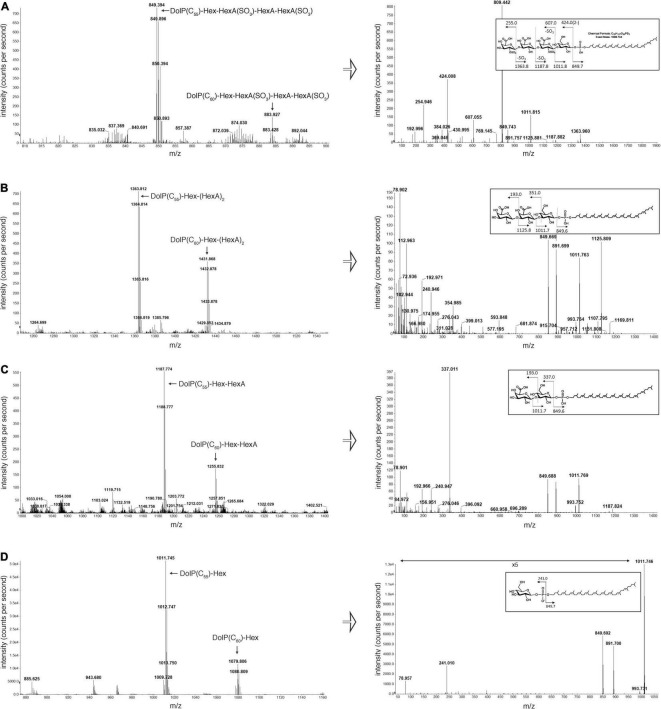
DolP-bound glycans are truncated in cells lacking *vng1062G, vng1066C* or *vng1067G*. **(A)** LC-ESI MS analysis of a total lipid extract from parent strain cells includes peaks corresponding to the doubly charged parent ion ([M-2H]^2–^) of C_55_- and C_60_-DolP modified by a hexose, a hexuronic acid and two sulfated hexuronic acids (left panel). Right panel: MS/MS profile of the tetrasaccharide-bearing C_55_-DolP moiety includes peaks predicted by the breakdown map of this glycosylated lipid (inset). MS/MS was performed on the doubly charged parent ion ([M-2H]^2–^) at m/z 849.39. **(B)** LC-ESI MS analysis of a total lipid extract from Δ*vng1062G* strain cells includes peaks corresponding to C_55_- and C_60_-DolP modified by a hexose and two hexuronic acids (left panel). Right panel: MS/MS profile of the trisaccharide-bearing C_55_-DolP moiety includes peaks predicted by the breakdown map of this glycosylated lipid (inset). MS/MS was performed on the doubly charged parent ion ([M-2H]^2–^) at m/z 681.7. **(C)** LC-ESI MS analysis of a total lipid extract from Δ*vng1066C* strain cells includes peaks corresponding to C_55_- and C_60_-DolP modified by a hexose and a hexuronic acid (left panel). Right panel: MS/MS profile of the disaccharide-bearing C_55_-DolP moiety includes peaks predicted by the breakdown map of this glycosylated lipid (inset). **(D)** LC-ESI MS analysis of a total lipid extract from Δ*vng1067G* strain cells includes peaks corresponding to C_55_- and C_60_-DolP modified by a hexose (left panel). Right panel: MS/MS profile of the hexose-bearing C_55_-DolP moiety includes peaks predicted by the breakdown map of this glycosylated lipid (inset). The arrow indicating ×5 in the MS/MS profile from the Δ*vng1067G* strain cells (bottom right panel) reflects magnification of the ion peaks in the corresponding region of the m/z values on the graph.

The N-linked tetrasaccharide decorating the S-layer glycoprotein is assembled on a DolP carrier (Lechner et al., 1985a; Vershinin et al., 2021). To assess the involvement of VNG1062G, VNG1066C, and VNG1067G in the assembly of this lipid-linked tetrasaccharide, the DolP pool was isolated from parent strain and Δ*vng1062G*, Δ*vng1066C*, and Δ*vng1067G* strain cells and examined by LC-ESI MS. Although tetrasaccharide-modified DolP was readily seen in the parent strain (the major DolP species modified by a linking hexose, a sulfated hexuronic acid, a hexuronic acid and a second sulfated hexuronic acid is shown (the [M-2H]^2–^ species are shown; exact mass of the C_55_ species is 1698.744 Da), rather than the more minor species methylated on the final hexuronic acid (see Figure 4, Vershinin et al., 2021; Figure 4A), DolP from the three mutant strains only contained precursors of this glycan. In Δ*vng1062G* strain cells, the most elaborate glycan detected attached to DolP contained a hexose and two hexuronic acids [modified DolP(C_55_) species, [M-H]^–^ ion, calculated *m/z* 1363.81, observed *m/z* 1363.81] (Figure 4B, left panel). At the same time, Δ*vng1066C* strain cells contained DolP modified only by a hexose and a hexuronic acid [modified DolP(C_55_) species, [M-H]^–^ ion, calculated *m/z* 1187.78, observed *m/z* 1187.77] (Figure 4C, left panel). Finally, the only modified DolP species in Δ*vng1067G* strain cells corresponded to the lipid modified by just a hexose [modified DolP(C_55_) species, [M-H]^–^ ion, calculated *m/z* 1101.74, observed *m/z* 1011.73] (Figure 4D, left panel). These assignments were confirmed when the MS/MS profiles of each modified DolP species were considered, as in each case, the expected breakdown fragments were detected (Figures 4A–D, right panels). An additional comparison of the MS profiles of sugar-charged DolP species from the parent and mutant strains is presented in Supplementary Figure 3.

In summary, given the effects of deleting *vng1062G*, *vng1066C*, and *vng1067G* on *Hbt. salinarum* N-glycosylation, these genes were re-named *agl27*, *agl26*, and *agl25*, respectively, according to the nomenclature used for naming genes involved in archaeal N-glycosylation and reflecting the order in which these glycosyltransferases participate in the assembly of the N-linked tetrasaccharide on a DolP carrier, which is then transferred to *Hbt. salinarum* glycoproteins (Eichler et al., 2013b).

### The Absence of Agl25, Agl26 or Agl27 Leads to Reduced Transcription of Genes Encoding a Sub-Set of Archaellins

**FIGURE 5:**
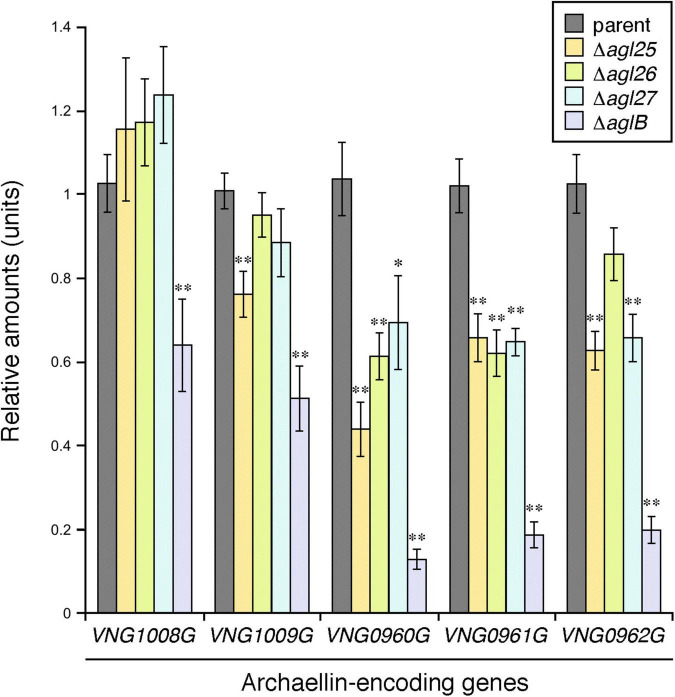
The transcription of genes encoding FlaB1, FlaB2, and FlaB3 is reduced in cells lacking Agl25, Agl26 or Agl27. The levels of cDNA, reverse-transcribed from mRNA isolated from parent, Δ*aglB*,Δ*agl25*, Δ*agl26*, and Δ*agl27* strain cells, were quantified by qRT-PCR using primers against *VNG1008G*, *VNG1009G*, *VNG0960G*, *VNG0961G*, and *VNG0962* (Table 1). Transcript levels of each gene were normalized to that of a housekeeping gene (*VNG0657G*) in the parent strain and corresponding deletion strain. The transcript levels of *VNG1008G*, *VNG1009G*, *VNG0960G*, *VNG0961G*, and *VNG0962* were then normalized to the levels measured in the parent strain. The values presented represent the average of triplicate repeats of four biological repeats ± SEM. *Statistically significant (Student’s *t* test) relative to the parent strain >95%. ^**^ Statistically significant relative to the parent strain >99%.

Having previously shown that *Hbt. salinarum* cells deleted of *aglB*, as such unable to N-glycosylate archaellins, showed reduced transcription of the genes encoding FlaA1 (*VNG1008G*), FlaA2 (*VNG1009G*), FlaB1 (*VNG0960G*), FlaB2 (*VNG0961G*), and FlaB3 (*VNG0962G*) (Zaretsky et al., 2019), the effects of *agl25*, *agl26*, and *agl27* deletion on the transcription of these five archaellin-encoding genes was tested by qRT-PCR. While the transcription of *VNG1008G* and *VNG1009G* was decreased as a result of such deletions, these effects were not significant for the most part. In contrast, highly significant reductions in the transcription of *VNG0960G*, *VNG0961G*, and *VNG0962G* were observed in the Δ*agl25*, Δ*agl26*, and Δ*agl27* strains (Figure 5). Finally, in agreement with an earlier report (Zaretsky et al., 2019), transcription of all five archaellin-encoding genes was very significantly reduced in the Δ*aglB* strain.

## Discussion

In 1976, the *Hbt. salinarum* S-layer glycoprotein provided the first example of N-glycosylation outside the Eukarya (Mescher and Strominger, 1976). Soon after, the same N-linked glycan was reported as also modifying archaellins in this haloarchaeon (Wieland et al., 1985). Additional efforts made at the time attempted to define the composition of this N-linked glycan and gain insight into its biosynthesis, despite the lack of genomic information and appropriate genetic tools. Although relatively little progress in understanding *Hbt. salinarum* N-glycosylation was made in the almost four decades since these pioneering studies were conducted, attention is once again focusing on how *Hbt. salinarum* performs this post-translational modification. Most recently, mass spectrometry was used to define the composition of the N-linked glycan, shown to be a tetrasaccharide comprising Hex-HexA(SO_3_)-HexA-HexA(SO_3_) (Vershinin et al., 2021). These efforts thus clarified earlier and often contradictory descriptions of this moiety (Wieland et al., 1980, 1983, 1985; Lechner et al., 1985b). Relatively recent efforts also proposed a series of *Hbt. salinarum* proteins as comprising the pathway recruited for assembly of this N-linked tetrasaccharide (Kandiba and Eichler, 2015). In the present report, the involvement of three of these proteins in *Hbt. salinarum* N-glycosylation was considered by addressing N-glycan composition in cells lacking each encoding gene. Specifically, it was revealed that in *Hbt. salinarum*, Agl25 (VNG1067G) is responsible for adding the first of the three hexuronic acids of the N-linked tetrasaccharide to DolP-Hex, Agl26 (VNG1066C) is responsible for adding the second hexuronic acid to DolP-Hex-HexA, and Agl27 (VNG1062G) adds the final hexuronic acid to the DolP-Hex-(HexA)_2_ precursor.

Previous efforts in which *Hbt. salinarum* cells lacking AglB were addressed demonstrated the importance of N-glycosylation for various aspects of *Hbt. salinarum* cell biology (Zaretsky et al., 2019). The present study extended these findings by addressing the importance of complete and proper N-glycosylation. It was shown here that in the Δ*agl25*, Δ*agl26*, and Δ*agl27* strains, motility and archaella levels were compromised. Specifically, motility was greatly decreased, if not absent in cells lacking Agl25 and Agl26, but much less so in cells lacking Agl27, relative to the parent strain. Similarly, although archaellins were synthesized in all three deletion strains, archaellin levels were seemingly very low in the Δ*agl25* and Δ*agl26* strains and only partially compromised in Δ*agl27* cells, again relative to the parent strain. The slightly improved motility of cells lacking Agl27 relative to those lacking Agl26 likely reflects partial reversion of the Δ*agl27* strain, rather than differential effects of deleting either glycosyltransferase-encoding gene. The impact of incomplete N-glycosylation on cell motility was also reported in the haloarchaeon *Hfx. volcanii* (Tripepi et al., 2012). Here, absence of AglJ, AglG, AglI or AglD, glycosyltransferases, respectively responsible for adding the first, second, third and fifth sugars of the N-linked pentasaccharide assembled in this species (Abu-Qarn et al., 2007; Yurist-Doutsch et al., 2008; Kaminski et al., 2010), resulted in a loss of motility. Some motility was detected in cells lacking AglE, responsible for adding the fourth sugar of the N-linked pentasaccharide (Abu-Qarn et al., 2008). In cells of the methanogen *Methanococcus voltae* lacking the glycosyltransferases AglC and AglK, reportedly involved in adding either the first and second or only the second sugar to the glycan N-linked to archaellins in this methanogen (Chaban et al., 2009; Larkin et al., 2013), no archaella or cell motility were observed (Chaban et al., 2009). *M. voltae* deleted of *aglA*, encoding the glycosyltransferase responsible for adding the final sugar of the N-linked glycan, were only able to assemble few archaella and were only weakly motile (Chaban et al., 2006). In *Methanococcus maripaludis*, where archaellins are modified by an N-linked tetrasaccharide (Kelly et al., 2009), the absence of the glycosyltransferases AglA, responsible for adding the third sugar, or AglL, responsible for adding the fourth sugar, did not prevent the assembly of archaella, yet impaired cell motility. In contrast, cells lacking AglO, responsible for adding the second sugar, were non-motile and failed to assemble archaella (VanDyke et al., 2009). Finally, in *Sulfolobus acidocaldarius*, a lack of the glycosyltransferase Agl16, responsible for adding the final glucose to the N-linked hexasaccharide assembled in this thermoacidophile, yielded cells displaying drastically reduced motility and no archaella (Meyer et al., 2013). Finally, the absence of Agl25, Agl26, and Agl27 compromised S-layer architecture, as reflected in the increased susceptibility of the S-layer glycoprotein in the deletion strains to proteolysis. A similar effect of compromised S-layer glycoprotein N-glycosylation on S-layer architecture was also seen in *Hfx. volcanii* (Tamir and Eichler, 2017). The N-linked glycans decorating *Hbt. salinarum* proteins, specifically the S-layer glycoprotein and archaellins, are in direct contact with the outside world. As such, N-glycan composition likely affects not only S-layer architecture and archaellum assembly and function but also other aspects of *Hbt. salinarum* cell biology. *Hbt. salinarum* represents one of the few archaeal models addressed at the systems-level (Brooks et al., 2014; Darnell and Schmid, 2015). The identification of Agl25, Agl26 and Agl27 as glycosyltransferases involved in *Hbt. salinarum* N-glycosylation, along with earlier identification of the oligosaccharyltransferase AglB (Zaretsky et al., 2019), thus provide the basis for considering N-glycosylation in systems biology-based studies. Indeed, the observation that the transcription of some but not all archaellin-encoding genes was compromised in cells lacking Agl25, Agl26 or Agl27 should encourage such efforts.

Identifying Agl25, Agl26, and Agl27 as three of the four glycosyltransferases involved in *Hbt. salinarum* N-glycosylation also offers novel insight into the N-glycosylation pathway in this organism. For instance, whereas the hexuronic acids at positions two and four of the DolP-bound and N-linked tetrasaccharide are sulfated (Vershinin et al., 2021), in Δ*agl26* and Δ*agl27* cells, in which only the first two and first three sugars of the tetrasaccharide are, respectively, found, the hexuronic acid at position two was not sulfated. This suggests that the hexuronic acid at position two (and possibly the hexuronic acid at position four) are only sulfated once the complete tetrasaccharide is assembled on the DolP carrier. This is apparently in contrast to what is thought to occur in other Archaea, specifically *S. acidocaldarius*, where Agl3 was shown to be a UDP-sulfoquinovose synthase and important for N-glycosylation, suggesting that in this species, sulfation occurs at the nucleotide-linked sugar level (Meyer et al., 2011). The absence of N-linked glycan sulfation, as well as the detection of precursors/breakdown products of the DolP- and Asn-bound disulfated tetrasaccharide presenting a single sulfate group on either the second or the fourth hexuronic acid, respectively, reported in our earlier efforts (Zaretsky et al., 2019; Vershinin et al., 2021) could reflect lability of sugar-bound sulfate groups. However, since sulfated breakdown products were detected by MS/MS of the tetrasaccharide attached to either DolP or protein targets (see Figure 4A and Supplementary Figure 2A), whereas no sulfation of the precursor glycans in the mutant strains was seen, despite the identical processing of parent and mutant strain samples, it is unlikely that sulfate group loss occurred in the present study. If sulfation of the first and third hexuronic acids of the tetrasaccharide N-linked to *Hbt. salinarum* glycoproteins indeed occurs only after all sugars are first added to the DolP carrier, then variability in the extent of such sulfation is conceivable, possibly in response to changes in growth conditions (Eichler, 2020). However, no changes in N-linked tetrasaccharide sulfation were noted when *Hbt. salinarum* were raised at three different NaCl concentrations (Vershinin et al., 2021). In addition, the present study may also offer further insight into the composition of the N-linked tetrasaccharide. If the N-linked tetrasaccharide indeed comprises a Glc and three GlcAs, all connected via 1–4 linkages, as previously reported (Lechner et al., 1985a), then it is not clear why *Hbt. salinarum* would require two different glycosyltransferases, i.e., Agl26 and Agl27, to combine the same two sugars. The possibilities that different hexuronic acids are joined by each enzyme and/or that different linkages are generated between the second and third and the third and fourth hexuronic acids await more detailed analysis of N-linked tetrasaccharide composition and architecture.

Finally, it was previously reported that the tetrasaccharide ultimately N-linked to selected target protein asparagine residues is methylated at the non-reducing end in the DolP-bound but not in the protein-bound stage. Whereas earlier studies assigned such methylation to a glucose found at the non-reducing end (Lechner et al., 1985b), more recent analysis reported methylation of the terminal hexuronic acid of the tetrasaccharide (Vershinin et al., 2021). These earlier efforts also reported such methylation to be essential for *Hbt. salinarum* N-glycosylation and proposed a role for such methylation either in the transfer of the DolP-glycan across the plasma membrane or as a marker recognized by the translocation machinery (Lechner et al., 1985b). However, the present study found that the DolP-bound tetrasaccharide precursors assembled in cells lacking Agl25, Agl26 or Agl27, and hence each lacking a methylated HexA in the fourth position, were delivered to target proteins. These observations argue that methylation of the DolP-bound glycan is not essential for *Hbt. salinarum* N-glycosylation nor for transfer of the DolP-bound glycan across the membrane. DolP-bound glycan methylation could instead increase the efficiency of either translocation of the DolP-bound glycan across the membrane or transfer of the glycan to target protein asparagine residues. However, given how methylated DolP-glycan accumulated in cells deleted of *aglB* (Vershinin et al., 2021), it is less likely that methylation is important for DolP-bound tetrasaccharide delivery across the membrane. Defining the methyltransferase responsible for DolP-bound glycan methylation or the demethylase responsible for reversing this reaction will help clarify this matter.

**FIGURE 6:**
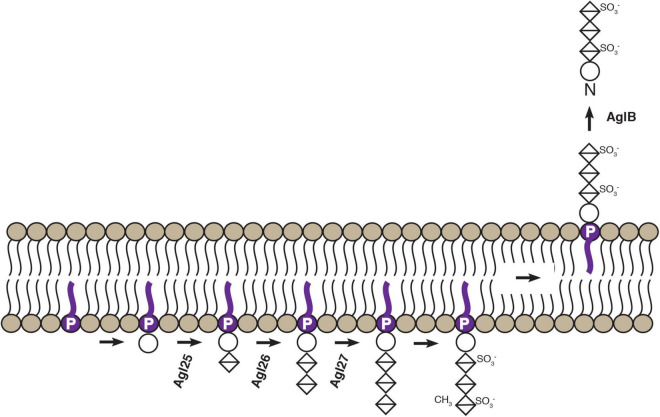
Schematic depiction of confirmed steps of an *Hbt. salinarum* N-glycosylation pathway. Based on the findings reported here and previously (Zaretsky et al., 2019; Vershinin et al., 2021), experimentally confirmed steps of the *Hbt. salinarum* pathway responsible for assembling and transfer of an N-linked tetrasaccharide are presented. DolP is shown in purple. Circles correspond to hexoses and crossed diamonds corresponds to hexuronic acids.

In summary, Agl25, Agl26, and Agl27 now join the oligosaccharyltransferase AglB as experimentally characterized components of the *Hbt. salinarum* N-glycosylation pathway (Figure 6). The results reported here will encourage renewed interest in the process and importance of N-glycosylation in *Hbt. salinarum*, where this post-translation modification was first described in Archaea (and indeed, outside the Eukarya) over four decades ago, after having been overlooked for far too long.

## Data Availability Statement

The original contributions presented in the study are included in the article/Supplementary Material, further inquiries can be directed to the corresponding author.

## Author Contributions

ZV and MZ performed the experiments. ZV, MZ, ZG, and JE analyzed the data. JE wrote the manuscript with contributions from all authors.

## Conflict of Interest

The authors declare that the research was conducted in the absence of any commercial or financial relationships that could be construed as a potential conflict of interest.

## Publisher’s Note

All claims expressed in this article are solely those of the authors and do not necessarily represent those of their affiliated organizations, or those of the publisher, the editors and the reviewers. Any product that may be evaluated in this article, or claim that may be made by its manufacturer, is not guaranteed or endorsed by the publisher.
