# Supplementary material for: Identifying Components of a Halobacterium salinarum N-Glycosylation Pathway
Source: Front Microbiol. 2021 Dec 2;12:779599. doi: 10.3389/fmicb.2021.779599 (PMC8674786; doi:10.3389/fmicb.2021.779599)
Supplement: Supplementary file 1 [file Data_Sheet_1.docx]

Supplementary Material

**Identifying components of a *Halobacterium salinarum* N-glycosylation pathway**

**Zlata Vershinin^1^, Marianna Zaretsky^1^, Ziqiang Guan^2^ and Jerry Eichler^1,^***

^1^Department of Life Sciences, Ben-Gurion University of the Negev, Beersheva, Israel

^2^Department of Biochemistry, Duke University Medical Center, Durham NC, USA

***Correspondence:** Prof. Jerry Eichler ([jeichler@bgu.ac.il](mailto:jeichler@bgu.ac.il))

**
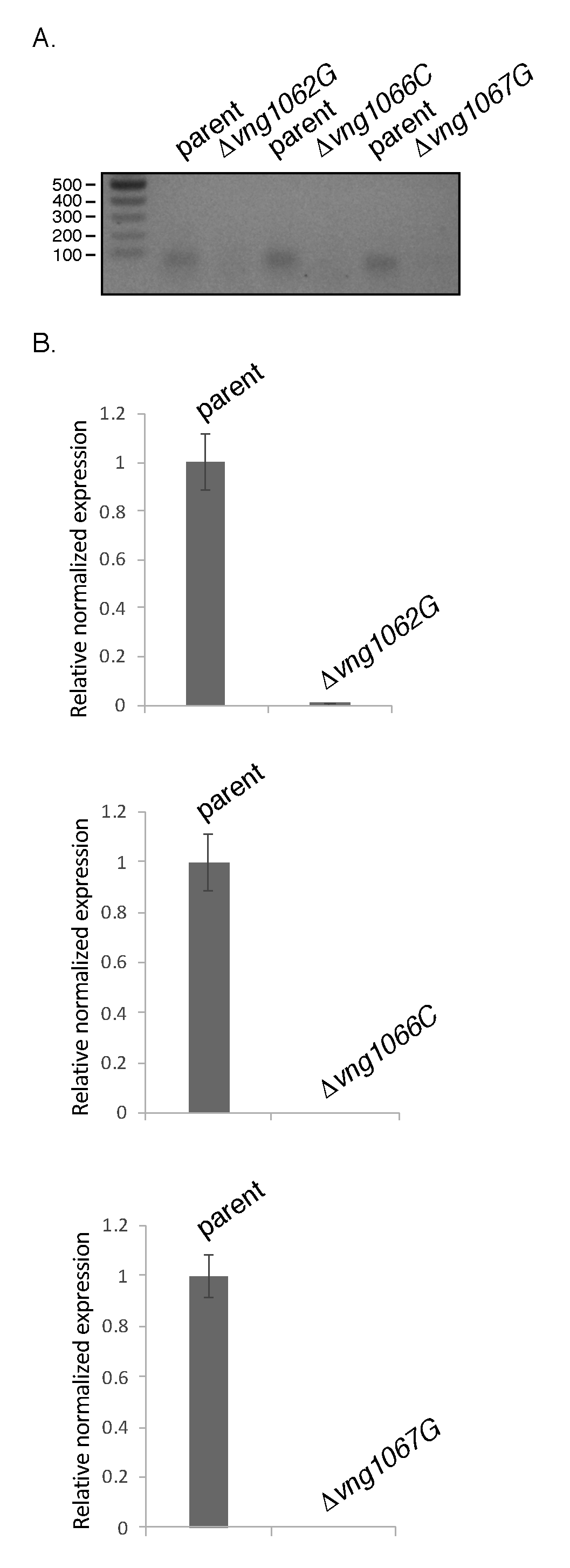
Supplementary Fig. S1 - Validation of gene deletions by qRT-PCR.** The levels of cDNA, reverse-transcribed from mRNA isolated from parent, Δ*vng1062G*, Δ*vng1066C* and Δ*vng1067G* strain cells, were quantified by qRT-PCR using the specific primers indicated in Table 1. **A.** qRT-PCR-amplified products (*VNG1062G* (83 bp), *VNG1066C* (128 bp) and *VNG1067G* (94 bp)) from parent (lanes 2, 4 and 6), Δ*vng1062G* (lane 3), Δ*vng1066C* (lane 5) and Δ*vng1067G* (lane 7) strain cells were analyzed on a 1% agarose gel. Markers of 100-500 bp are shown on the left (lane 1). **B.** The level of mRNA expression for each gene of interest was normalized to that of a housekeeping gene (*VNG0657G*) in the parent strain and corresponding deletion strain. The level of mRNA expression of each gene of interest was then normalized to the level measured in the parent strain, taken as 1.0. The values presented represent the average of four repeats ± SEM.

**Supplementary Fig. S
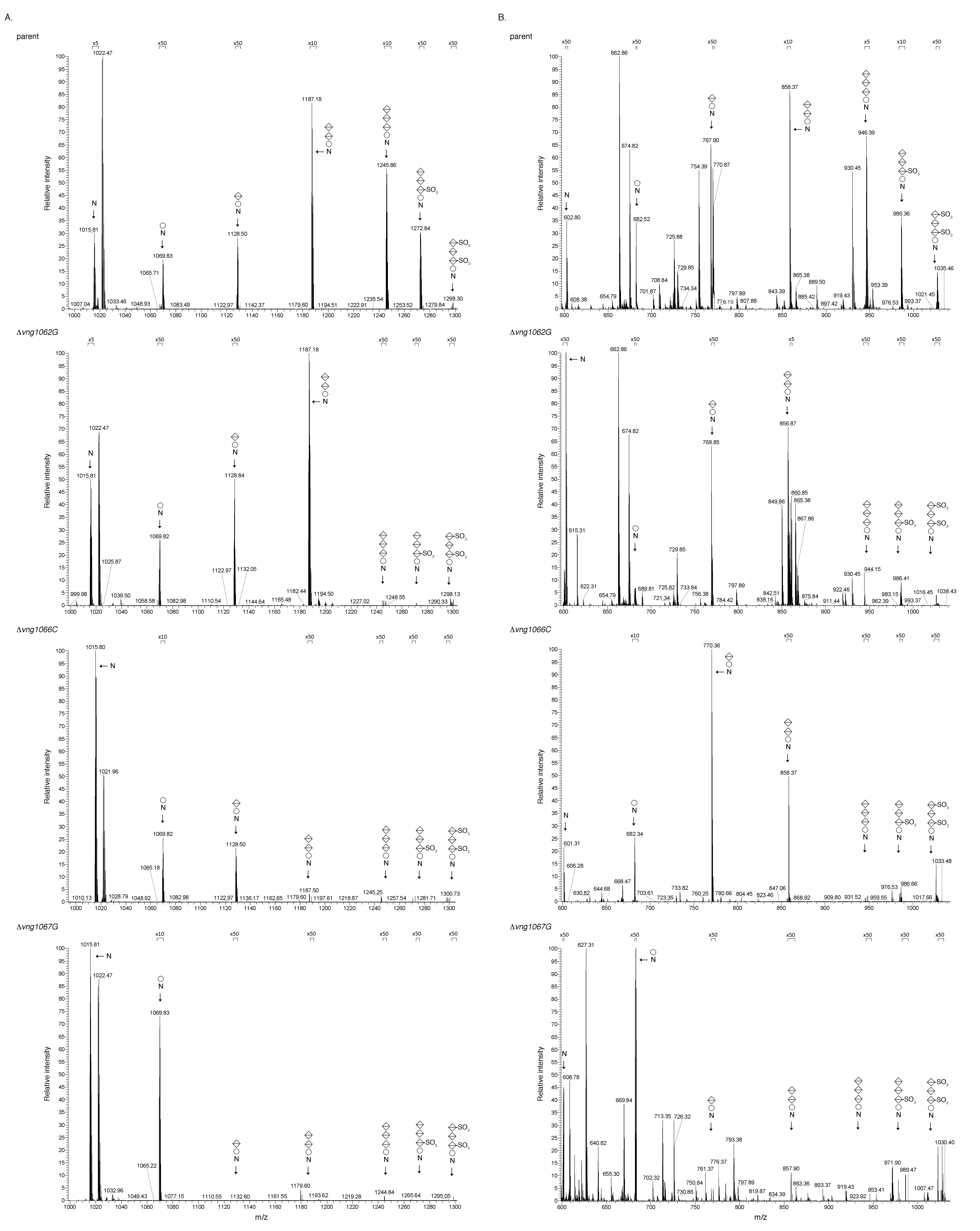
2**

**Supplementary Fig. S2 - MS profiles of S-layer glycoprotein- and archaellin-derived glycopeptides from parent and mutant strain cells.** MS profiles covering the m/z region that includes non-modified to tetrasaccharide-modified protease-generated (**A.**) S-layer glycoprotein Asn-479-containing glycopeptide and (**B.**) the QAAGADNINLSK glycopeptide derived from the archaellins FlaA1, FlaA2 and FlaB2 from parent (top row), Δ*vng1062G* (second row), Δ*vng1066C* (third row) and Δ*vng1067G* (bottom row) strain cells. The positions of the peptides modified by the complete N-linked disulfated tetrasaccharide and precursors thereof are indicated in each panel. N corresponds to the peptide, the circles correspond to hexoses, the split diamonds corresponds to hexuronic acids and SO_3_ corresponds to a sulfate group. The regions of the MS profiles marked with x5, x10 and x50 reflect magnification of the m/z values of the ion peaks in the corresponding region of the profile.

**
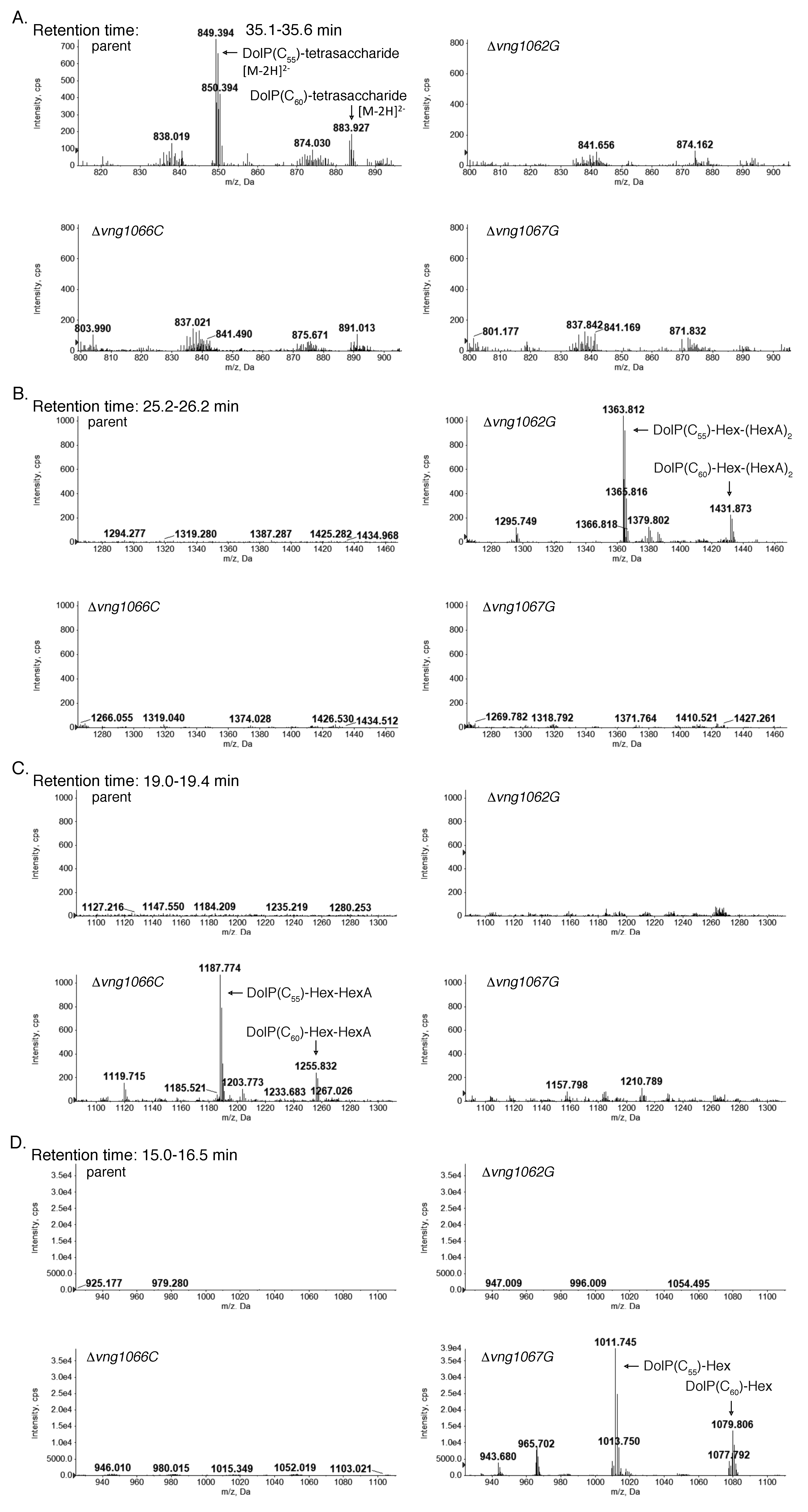
Supplementary Fig. S3**

**Supplementary Fig. S3 - MS profiles of DolP charged with a tetrasaccharide and its precursors.** Profiles obtained following normal phase LC-ESI MS analysis of DolP-based species retained for the indicated times. Each set of panels presents the profile obtained at the retention times when **A.** tetrasaccharide-modified DolP, **B.** trisaccharide-modified DolP, **C.** disaccharide-modified DolP and **D.** monosaccharide-modified DolP appeared from parent, Δ*vng1062G*, Δ*vng1066C* and Δ*vng1067G* strain cells, as indicated.
